# A Natural System of Chromosome Transfer in *Yersinia pseudotuberculosis*

**DOI:** 10.1371/journal.pgen.1002529

**Published:** 2012-03-08

**Authors:** Biliana Lesic, Mohamed Zouine, Magaly Ducos-Galand, Christèle Huon, Marie-Laure Rosso, Marie-Christine Prévost, Didier Mazel, Elisabeth Carniel

**Affiliations:** 1 Yersinia Research Unit, Institut Pasteur, Paris, France; 2 Unité Plasticité du Génome Bactérien, Institut Pasteur, CNRS UMR 3525, Paris, France; 3 Plateforme de Microscopie Ultrastructurale, Institut Pasteur, Paris, France; Universidad de Sevilla, Spain

## Abstract

The High Pathogenicity Island of *Yersinia pseudotuberculosis* IP32637 was previously shown to be horizontally transferable as part of a large chromosomal segment. We demonstrate here that at low temperature other chromosomal loci, as well as a non-mobilizable plasmid (pUC4K), are also transferable. This transfer, designated GDT4 (Generalized DNA Transfer at 4°C), required the presence of an IP32637 endogenous plasmid (pGDT4) that carries several mobile genetic elements and a conjugation machinery. We established that cure of this plasmid or inactivation of its sex pilus fully abrogates this process. Analysis of the mobilized pUC4K recovered from transconjugants revealed the insertion of one of the pGDT4–borne ISs, designated IS*Yps1*, at different sites on the transferred plasmid molecules. This IS belongs to the IS*6* family, which moves by replicative transposition, and thus could drive the formation of cointegrates between pGDT4 and the host chromosome and could mediate the transfer of chromosomal regions in an Hfr-like manner. In support of this model, we show that a suicide plasmid carrying IS*Yps1* is able to integrate itself, flanked by IS*Yps1* copies, at multiple locations into the *Escherichia coli* chromosome. Furthermore, we demonstrate the formation of RecA-independent cointegrates between the IS*Yps1*-harboring plasmid and an IS*Yps1*-free replicon, leading to the passive transfer of the non-conjugative plasmid. We thus demonstrate here a natural mechanism of horizontal gene exchange, which is less constrained and more powerful than the classical Hfr mechanism, as it only requires the presence of an IS*6*-type element on a conjugative replicon to drive the horizontal transfer of any large block of plasmid or chromosomal DNA. This natural mechanism of chromosome transfer, which occurs under conditions mimicking those found in the environment, may thus play a significant role in bacterial evolution, pathogenesis, and adaptation to new ecological niches.

## Author Summary

All living species have the capacity to evolve in order to adapt to new and often hostile conditions. Horizontal gene transfer is a major route for rapid bacterial evolution. Some clearly identified mobile genetic elements (plasmids, phages, etc.) are by essence exchanged between bacteria. However, the mechanisms generating the bacterial core genome diversity are much less understood. In this study we have characterized in *Y. pseudotuberculosis*, a natural bacterial pathogen causing mesenteric lymphadenitis and enteritis, a mechanism of horizontal gene exchange that conveys the transfer of virtually any piece of chromosomal or plasmid DNA to a new bacterial host. This generalized mechanism of DNA transfer is optimal when the bacteria encounter conditions that might resemble those they met in their natural ecological niches. We demonstrate that this transfer mechanism is extremely powerful, as the presence on a conjugative replicon of an insertion sequence having a low specificity of insertion and transposing through replicative transposition is sufficient to drive the horizontal transfer of virtually any piece of chromosomal or episomal DNA. As such, this mechanism is much less constrained than the classical Hfr mechanism described in laboratory *E. coli* and could be used by a wide variety of bacterial species for gene exchange and evolution.

## Introduction

Horizontal gene transfer (HGT) is a driving force for bacterial evolution, as it allows the dispersion of adaptive loci between closely related and also phylogenetically distant bacterial species. Well-characterized mobile genetic elements such as conjugative plasmids, transposons, Integrative conjugative elements (ICE), pathogenicity islands (PAI), or phages are associated with HGT of specific adaptive functions (antibiotic resistance, virulence, metabolic pathways) and participate to genome plasticity. However, exchanges of chromosomal regions that form the core genome and are not part of the mobile genetic pool are also observed. While their importance in bacterial evolution and speciation is now well established, the underlying mechanisms are often loosely described and remain hypothetical in many cases.

The Gram-negative enteropathogen *Yersinia pseudotuberculosis* carries a PAI termed High Pathogenicity Island (HPI) [1], which encodes the siderophore yersiniabactin [2]. The fact that this island is mobile within the genome of its host strain [3], and is present and often conserved both in terms of genetic organization and nucleotide sequence in various bacterial genera such as *Escherichia coli* (various pathotypes), *Klebsiella* or *Citrobacter* [4], suggested that it may have retained its ability to be horizontally transmitted to new bacterial hosts. Indeed, we evidenced the transfer of the HPI between natural *Y. pseudotuberculosis* isolates [3]. This phenomenon was observed only when the bacteria were incubated at low temperature (optimal at 4°C) and in broth, and was more efficient in an iron-poor medium [5]. However, this transfer did not require the integration/excision machinery encoded by the HPI, was RecA-dependent in the recipient strain, and involved not only the HPI but also adjacent sequences encompassing at least 46 kb of chromosomal DNA [3]. Similar results were recently obtained for the HPI of natural *Escherichia coli* isolates, using a multi locus sequence typing approach. The *E. coli* HPI was found to have been acquired simultaneously with the chromosomal flanking regions of the donor strains [6], indicating again that the island was transmitted as part of a larger chromosomal region. This phenomenon is not restricted to the HPI and to enterobacteria since it has been recently reported that movement of the *Enterococcus faecalis* PAI was invariably accompanied by transfer of flanking donor chromosome sequences [7].

The aim of this work was to characterize the mechanisms underlying horizontal chromosomal gene transfer in *Y. pseudotuberculosis*. We describe here a natural system of conjugative transfer, which may be used by a wide variety of bacterial species for gene exchanges, and which may represent a driving force for bacterial evolution.

## Results

### Generalized transfer of chromosomal and plasmid DNA in *Y. pseudotuberculosis* IP32637

**Figure 1:**
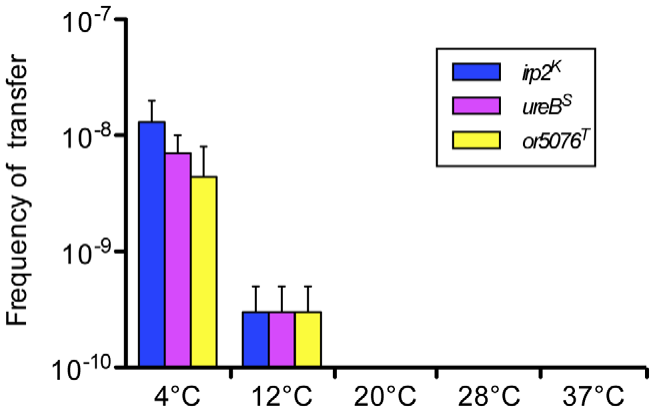
Transfer frequencies at various temperatures of three distantly located chromosomal loci. The donor 637-*irp2^K^-ureB^S^-5076^T^* and recipient 637ΔHPI-Nal^R^ strains were co-incubated in LB-αα' with agitation. Transfer frequency was calculated as the number of Nal^R^ (or Kan^R^, Spe^R^, Tmp^R^) and Rif^S^ transconjugants per Rif^R^ donor cells. Shown are mean values of transfer frequencies (vertical bars) and standard error of the mean (sem, vertical lines) of two independent experiments at each temperature. Mean transfer frequencies (±sem) at 4°C: 1.3(±0.7)×10^−8^ for *irp2* ^K^, 0.7(±0.3)×10^−8^ for *ureB* ^S^ and 0.4(±0.4)×10^−8^ for *or5075* ^T^, and at 12°C: 0.03(±0.02)×10^−8^ for the three loci. Transfer frequencies at temperatures ≥20°C were systematically below the detection limit (10^−10^).

Since we did not know whether the lateral transfer process previously observed was limited to the region encompassing the HPI or could involve any portion of the chromosome, two other loci (*ureB* and *or5076*) were labeled with a spectinomycin (Spe) and trimethoprim (Tmp) resistance cassette, respectively. These two genes were chosen because, based on the IP32953 sequence, they are predicted to be separated from each other and from the HPI (tagged with a kanamycin (Kan) cassette in the *irp2* gene) by at least 1.5 Mb of chromosomal DNA (Figure S1). Moreover, the *ureB* gene, which is part of the urease locus, and *or5076*, encoding a putative toxin transporter [8] are not predicted to be involved in DNA transfer. After co-incubation of the donor 637-*irp2^K^-ureB^S^-5076* ^T^ and recipient 637ΔHPI-Nal^R^ strains (Table 1) under conditions (4 days at 4°C in LB-αα' with shaking) that we previously found to be optimal for HPI transfer [3], recipient strains having acquired either the *irp2^K^* (Nal^R^, Kan^R^, Rif^S^), *ureB^S^* (Nal^R^, Spe^R^, Rif^S^) or *or5076^T^* (Nal^R^, Tmp^R^, Rif^S^) antibiotic resistances were obtained. Acquisitions of the corresponding tagged loci were checked by PCR (Figure S1). Transfer frequencies were of the same magnitude for the three antibiotic-tagged loci (≈10^−8^, Figure 1). None of the transconjugants obtained had simultaneously acquired two of the antibiotic-tagged loci, indicating that the sizes of the chromosomal fragments transferred were inferior to 1.5 Mb.

**Table 1:** *Y. pseudotuberculosis* strains and plasmids used for DNA transfer experiments.

Strains	Characteristics	Antibiotic resistance	Source or reference
**IP32637 and derivatives**			
IP32637	Wild type, pYV, pGDT4, serotype I	None	Institut Pasteur
637-Rif^R^	Spontaneous Rif^R^ mutant of IP32637	Rif	[3]
637-Nal^R^	Spontaneous Nal^R^ mutant of IP32637	Nal	This study
637ΔHPI-Nal^R^	Spontaneously Nal^R^ and HPI deleted strain IP32637	Nal	[3]
637ΔHPI-Rif^R^	Spontaneously Rif^R^ and HPI deleted strain IP32637	Rif	This study
637-*irp2^K^-ureB^S^*	Insertion of the Kan and Spe cassettes into *irp2* and *ureB*, respectively in 637-Rif^R^	Rif, Kan, Spe	This study
637-*irp2^K^-ureB^S^-5076^T^*	637-*irp2^K^-ureB^S^* tagged with Tmp (*5076^T^*), Rif^R^	Rif, Kan, Spe, Tmp	This study
637-*irp2^K^-ureB^S^*(pGDT4^T^)	Insertion of the Tmp cassette into pGDT4 of 637-*irp2^K^-ureB^S^*	Rif, Kan, Spe, Tmp	This study
637(pUC4K)	Introduction of pUC4K into 637-Rif^R^	Rif, Kan	This study
637(pUC4K, pGDT4Δ*pil*)	Replacement of a portion of the *pil* locus by a Tmp cassette in strain 637(pUC4K)	Rif, Kan, Tmp	This study
IP32637c	IP32637 cured of pYV and pGDT4	None	[9]
637c-Nal^R^	Spontaneous Nal^R^ mutant of IP32637c	Nal	This study
637c-Rif^R^	Spontaneous Rif^R^ mutant of IP32637c	Rif	This study
637c-*irp2^K^*	Insertion of Kan into the *irp2* gene of IP32637c-Rif^R^	Rif, Kan	This study
637c-*ureB^S^*	Insertion of Spe into the *irp2* gene of IP32637c-Rif^R^	Rif, Kan, Spe,	This study
**IP32953 and derivatives**			
IP32953	Wild type, pYV, pYptb32953, serotype I	None	Institut Pasteur
953-Rif^R^	Spontaneous Rif^R^ mutant of IP32953	Rif	This study
953-Nal^R^	Spontaneous Nal^R^ mutant of IP32953	Nal	[3]
953-*ureB^S^*(pGDT4)	A 953-Nal^R^ transconjugant that has acquired *ureB^S^* and pGDT4 from 637-*irp2^K^-ureB^S^*	Spe, Nal	This study
953-*irp2^K^-ureB^S^*	Insertion of the Kan and Spe cassettes into *irp2* and *ureB*, respectively in 953-Rif^R^	Rif, Kan, Spe	This study
**IP32777 and derivatives**			
IP32777	Wild type, serotype I	None	Institut Pasteur
777-Rif^R^	Spontaneous Rif^R^ mutant of IP32777	Rif	This study
777-*irp2^K^-ureB^S^*	Insertion of the Kan and Spe cassettes into *irp2* and *ureB*, respectively in 777-Rif^R^	Rif, Kan, Spe	This study
***E. coli*** ** and derivatives**			
ω7249	Allows pSW23T replication and transfer	Kan, Erm	[57]
ω7249(pSW*Yps1.1*)	ω249 harboring pSW*Yps1.1*	Cm	This study
ω4826	*recA* derivative of MG1655	Tc	[57]
ω4826::pSW*Yps1.1*	ω4826 transconjugant with chromosomal insertion of pSW*Yps1.1*	Cm	This study
pi3	*pir*+, R388	Tmp, Erm	[16]
pi3(R388, pSW*Yps1.2*)	pi3 carrying R388 and pSW*Yps1.2*	Tmp, Cm	This study
ω826(R388::pSW*Yps1.2*)	ω826 transconjugant harboring R388::pSW*Yps1.2* cointegrates	Tmp, Cm	This study

Plasmids			
pUC4K	High copy number	Kan	Amersham
pUC18	High copy number	Amp	Amersham
pcDNA-2.1	cloning vector, ColEI	Amp	Invitrogen
pSW25	*oriT*, *ccdB*	Spe	[52]
pGP704-*dfr*	*dfr* amplified from pNot::Ptac-*dfrB1*	Tmp, Amp	[3]
pKOBEG-*sacB*	Ts, low copy number, *sacB*, Cm^R^	Cm	[54]
pGDT4^T^	Insertion of a Tmp cassette by allelic exchange with the pGDT4-4kb segment	Tmp	This study
pSW23T	Suicide, mobilizable plasmid, oriV_R6K_, oriT_RP4_	Cm	[16]
pSW*Yps1.1*	Insertion of the region encompassing IS*Yps1* into pSW23T	Cm	This study
pSW23	Suicide, non-mobilizable plasmid, oriV_R6K_	Cm	[16]
pSW*Yps1.2*	Insertion of IS*Yps1* from pSW23T into pSW23	Cm	This study
R388	Conjugative, IncW plasmid	Tmp	F. de La Cruz
R388::pSW*Yps1.2*	Cointegrate between R388 and pSW*Yps1.2*	Tmp, Cm	This study

We previously showed that horizontal transfer of the HPI occurs only at low temperatures [3]. The same temperature dependency was observed for *ureB^S^* and *or5076^T^*: transfer of each of the three antibiotic-tagged loci was detected only when the donor and recipient strains were co-incubated at temperatures below 20°C (Figure 1), and was more efficient at 4°C than at 12°C (≥13 fold higher), as previously observed for *irp2^K^*. Therefore, distantly located chromosomal loci can be transferred with similar efficiencies and temperature regulations.

Whether this transfer mechanism could also mediate horizontal transmission of episomal molecules was addressed by introducing the non-conjugative and non-mobilizable plasmid pUC4K (Kan^R^) into the donor 637-Rif^R^. Using the defined optimal growth conditions, transfer of pUC4K from the 637(pUC4K) to the recipient 637ΔHPI-Nal^R^ was obtained and confirmed by PCR with primers 210A/210B (Table S1). This transfer occurred at a frequency of 2.3 (±0.4)×10^−7^, which is at least 10 times higher than that of chromosomal loci. Therefore, the process of DNA transfer is not limited to chromosomal DNA but can also involve plasmid molecules.

Altogether our results demonstrate the existence of a mechanism that potentially allows transfer of any chromosomal or episomal DNA molecule at low temperature. This mechanism was thus named GDT4 (for Generalized DNA Transfer at 4°C).

### IP32637 harbors a plasmid involved in GDT4

The capacity of other *Y. pseudotuberculosis* strains to mediate GDT4 was studied by tagging the IP32953 and IP32777 strains with both a Kan and Spe cassettes inserted into the *irp2* and *ureB* loci, respectively (Table 1). When these two recombinant strains were used as donors, no IP32637 transconjugants having acquired either *irp2^K^* or *ureB^S^* were obtained, indicating that GDT4 is not a property common to the entire *Y. pseudotuberculosis* species.

Strain IP32637 has the peculiarity of harboring an extra high molecular weight (≥100 kb) plasmid [9]. The role of this additional plasmid in chromosomal transfer was assessed by comparing GDT4 in IP32637 and its IP32637c plasmid-cured derivative [9]. Two tagged donor strains, 637c-*irp2^K^* and 637c-*ureB^S^* (Table 1), were generated and co-incubated with the 953-Nal^R^ recipient. No transconjugants were obtained, indicating a role of this plasmid in DNA transfer. The high molecular weight plasmid was thus designated pGDT4.

pGDT4 does not appear to be ubiquitous in the species *Y. pseudotuberculosis* as the genome sequences of IP32953 and of other *Y. pseudotuberculosis* strains available in databases did not evidence the presence of this plasmid. To get an insight into the frequency of pGDT4 carriage in this species, a 4 kb *Hin*dIII fragment of this episome, designated pGDT4.*seq* was cloned into pUC18, sequenced, and used to design primers (358A/B) for PCR screening. The analysis of a panel of 39 *Y. pseudotuberculosis* strains of serotypes I to V (Table S2) for the presence of the pGDT4 sequence identified two isolates (IP32699 and IP30215) that gave a PCR product of the expected size (Table S2). Both strains contained high molecular weight episomes whose *Hin*dIII-digestion patterns yielded some restriction fragments with a size similar to those of pGDT4, but the overall profiles of the three episomes were different (data not shown). Therefore, the plasmids found in IP32699 and IP30215 probably share some regions with pGDT4, but they are not identical to this plasmid. Since *Yersinia pestis* is a recent descent of *Y. pseudotuberculosis* [10], we also screened by PCR a panel of 51 strains of *Y. pestis* belonging to the three classical biovars (Antiqua, Medievalis and Orientalis) for the presence of the pGDT4-borne sequence. None of the strains tested yielded an amplification product (Table S2), suggesting the absence of vertical or horizontal transmission of pGDT4 to *Y. pestis*.

### pGDT4 is transferable

Plasmid analysis of transconjugants resulting from the co-incubation of the 637-*irp2^K^-ureB^S^* donor strain with the 637c-Nal^R^ recipient revealed that about half of them had acquired pGDT4 together with the chromosomal *irp2* (8/20) or *ureB* (11/20) tagged region, thus indicating that pGDT4 is also transferable.

**Figure 2:**
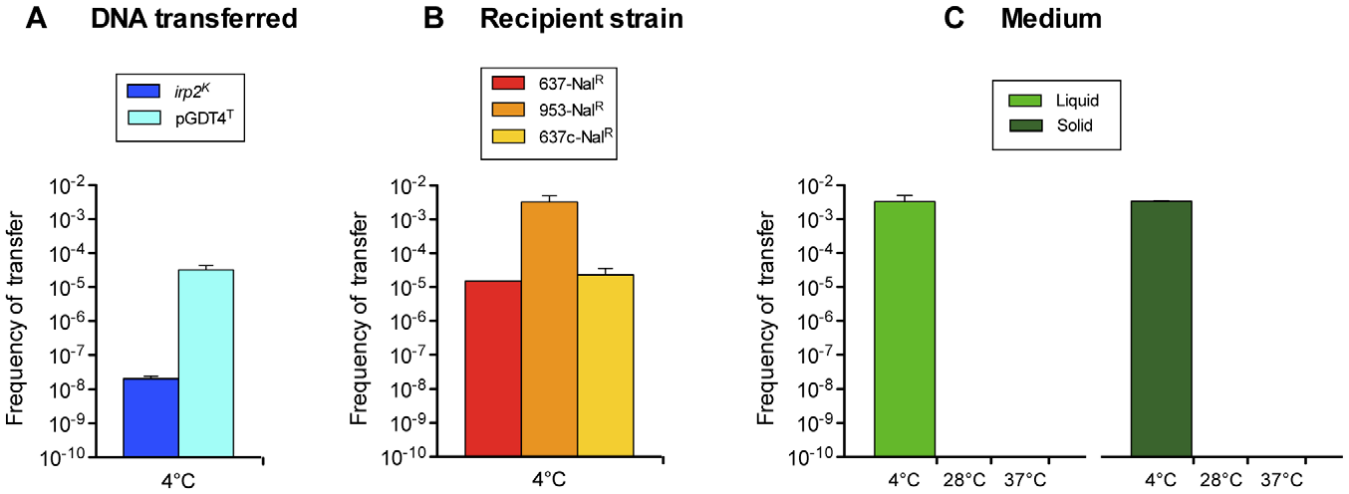
Conjugative properties of pGDT4. (A) Comparison of the efficiency of transfer of pGDT4 and the *irp2^K^* chromosomal locus. The donor 637-*irp2^K^-ureB^S^*(pGDT4^T^) and the recipient 637-Nal^R^ were co-incubated at 4°C in LB-αα' and independent experiments were performed 4 to 5 times. (B) Efficiency of transfer of pGDT4 to various recipient strains. The donor 637-*irp2^K^-ureB^S^*(pGDT4^T^) was co-incubated at 4°C with either 637-Nal^R^, 953-Nal^R^ or 637c-Nal^R^. The results of two independent experiments were combined. (C) Efficiency of transfer of pGDT4 at various temperatures in liquid and on solid media. The experiments were performed twice independently. Shown are mean values of transfer frequencies (vertical bars) and standard error of the mean (sem, vertical lines). The detection limit was 10^−10^.

To further study pGDT4 transfer capacity, the plasmid was labeled by allelic exchange of the pGDT4-4kb segment with a Tmp cassette. When the 637-*irp2^K^-ureB^S^* donor strain carrying the tagged pGDT4^T^ was co-incubated with the 637-Nal^R^ recipient, transconjugants harboring pGDT4^T^ were obtained with a frequency approximately 10^3^ times higher than that of chromosomal genes (Figure 2A and Table S3). The transfer frequency of pGDT4^T^ increased 200 fold when 953-Nal^R^ instead of 637-Nal^R^ was used as a recipient (Figure 2B and Table S3), indicating that properties inherent to the recipient cells may influence their capacity to take up pGDT4. The difference in the ability of the two strains to acquire this plasmid could not be explained by a mechanism of surface exclusion, as the frequency of transfer of pGDT4^T^ to IP32637 harboring or not harboring a resident pGDT4 was similar (Figure 2B and Table S3). Some unidentified intrinsic properties of the recipients such as a difference in their restriction/modification systems may be responsible for this difference. As observed with chromosomal DNA, no transfer of pGDT4^T^ was detected when the bacteria were mated at temperatures ≥28°C (Figure 2C and Table S3). However, in contrast to chromosomal DNA [3], pGDT4^T^ was transferable on nitrocellulose filters, at frequencies similar to those observed in a liquid medium (Table S3), but again, only at low temperature (Figure 2C). Therefore, transfer of pGDT4 is also temperature-dependent, but in contrast to chromosomal genes, it occurs at much higher frequencies, and both in liquid and on solid media.

Since the presence of pGDT4 is required for transfer, we wondered whether its presence could confer GDT4 properties to a strain that is naturally unable to mediate chromosomal transfer. For this purpose, a 953-*ureB^S^* transconjugant that had acquired pGDT4 simultaneously with chromosomal genes was used as donor and co-incubated with a 637ΔHPI-Rif^R^ recipient. While the parental 953-Nal^R^ strain was unable to transfer chromosomal DNA, the 953-*ureB^S^*(pGDT4) transconjugant gained the capacity to retransfer the acquired *ureB^S^* locus, though with a frequency 10 times lower (10^−9^) than that observed during the first transfer. These results further point at pGDT4 as a key element in the mechanism of chromosomal transfer.

### Sequence analysis of pGDT4

**Figure 3:**
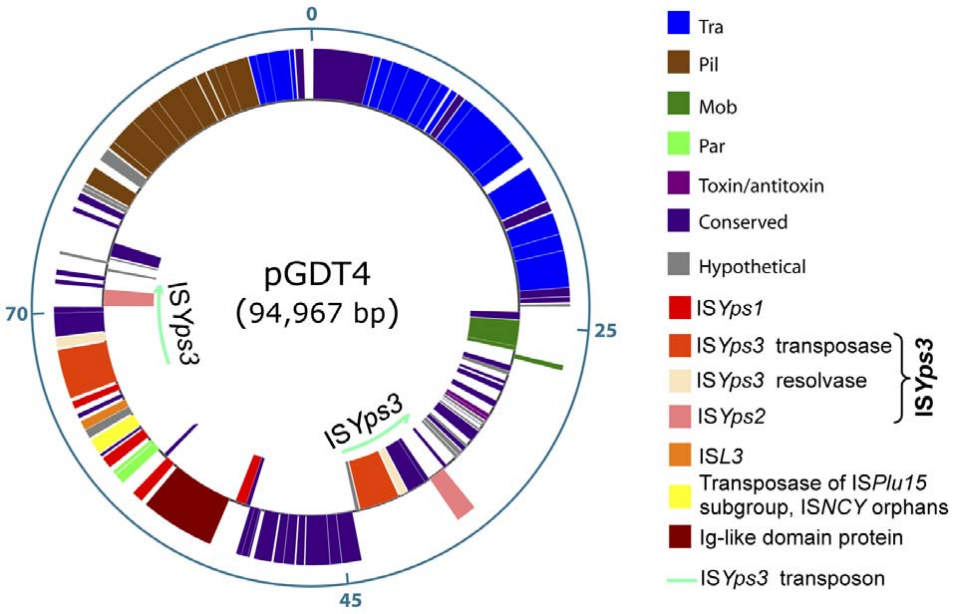
Genetic map of pGDT4.

To determine whether pGDT4 could encode its own transfer machinery, the plasmid was sequenced (EMBL accession number FM178282). The schematic map of the 94,967 bp circular plasmid molecule is shown on Figure 3. Of the 102 predicted coding sequences (cds) identified on pGDT4, 74 had homologs in databases (Table S4). Four major functional groups of genes were delineated on pGDT4:

1. A DNA fragment of ≈44 kb (pGDT4_0086-0024) carried genes predicted to be involved in conjugative transfer. Overall, these genes showed the closest similarity with those of the 153 kb conjugative plasmid pADAP of *Serratia entomophila* [11] that encode proteins involved in mating pair formation (MPF), DNA transfer and post conjugative replication. The pGDT4 MPF belongs to the MPF_I_ class, according to the most recent classification, for which R64 is the paradigm [12]. Interestingly, we found that in pGDT4, *traX* and *traY* are fused in a single *traXY* gene, while they are separated but adjacent in the related pADAP MPF operon. This is the first report of such organization, which seems to be functional as transfer of pGDT4 is occurring at reasonable rates. The classification of conjugative plasmids based on their encoded relaxase has recently emerged as powerful and meaningful in terms of plasmid physiology [13], [14]. The pGDT4 MobA relaxase is likely encoded by pGDT4_0022. This putative relaxase and its closest homolog, the protein pADAP_128 from pADAP, both fall in the MOB_P13_ class [14]. Proteins encoded by its neighboring genes, pGDT4_0023 and 0024, likely correspond to the relaxosomal accessory proteins MobB and MobC that act together with MobA in this family. Usually, in this type of *mobABC* backbone, the oriT is located between *mobC* (pGDT4_0024) and *mobB* (pGDT4_0023), as demonstrated for instance in pADAP [11].
2. The second group of genes gathered loci involved in plasmid stabilization and replication such as anti-restriction and toxin-antitoxin genes (pGDT4_0028-0031), the *parFG* operon (pGDT4_0062/0063, Table S4), encoding a partition system of type I (Walker type ATPase), and a replication initiation protein of the IncFII_RepA family (pGDT4_0081, Table S4). This replication protein is highly related to the RepA protein encoded by the *Yersinia enterocolitica* plasmid pYVe8081 [15], but this plasmid is otherwise unrelated to pGDT4 and has, for example, a partition system from a different family (SopAB).
3. The third group corresponded to mobile genetic elements, mostly transposases and insertion sequences (IS), which are commonly encountered on plasmids. pGDT4 carries one IS*L3* transposon, one uncharacterized IS (IS*NCY*) with a transposase of the IS*Plu15* subgroup belonging to the IS*NCY* orphans in the IS repository ISfinder, four copies (three complete and one truncated) of a new IS*6* family member (ISY*ps1*), and two copies of the transposon IS*Yps3* (Figure 3 and Table S4). The latter is a 7.3 kb Tn*3*-like transposon flanked by 34 bp inverted repeats and contains the IS*Yps3* transposase and resolvase, as well as IS*Yps2*, an IS*110* family member. None of the IS present on the *Y. pseudotuberculosis* IP32953 chromosome [8] were present on pGDT4 and conversely, no sequences homologous to the pGDT4-borne IS: IS*Yps1* (primer pair 727A/B), IS*NCY* (729A/B), IS*Yps2* (730A/B), IS*L3* (731A/B) and IS*Yps3* (732A/B) were detected by PCR in the IP32637 genome (data not shown).
4. The remaining genes included a large proportion of putative coding sequences of unknown functions, and a few loci with predictable functions, of which some shared homology with *Y. pseudotuberculosis* chromosomal genes (Table S4). The most remarkable example was the putative product of pGDT4_0059, which shared 41% amino acid identities with YPTB3789, a chromosomal Ig-like domain protein [16].

### pGDT4–mediated generalized DNA transfer

Since pGDT4 carries a large set of genes predicted to be involved in conjugative transfer, we wondered whether this pGDT4-specific mobility function mediates GDT4. To investigate this potential role, the Mpf function was inactivated by allelic exchange of a large portion of the *pil* region (from *pilL* to *pilV*) of pGDT4 with a Tmp cassette in 637(pUC4K). After co-incubation of the resulting strain 637(pUC4K, pGDT4Δ*pil*) with the 953-Nal^R^ recipient, no transconjugants having acquired pUC4K were obtained, indicating that the pilus-encoding region of pGDT4 is required for generalized DNA transfer.

**Figure 4:**
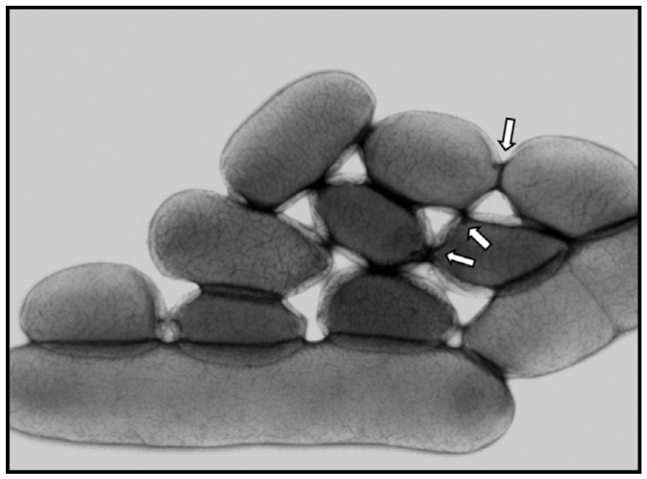
Electron microscopy of IP32637 grown at 4°C in LB with agitation. White arrows point at bridge-like structures.

As the Mpf region is predicted to encode a conjugative machinery, GDT4 most likely occurs by a mechanism of conjugation. To rule out other possible mechanisms of transfer, DNAse was added to the medium during the co-incubation period. The transfer frequency of the *irp2* locus from the donor 637-*irp2^K^-ureB^S^* to the recipient 637ΔHPI-Nal^R^ was not affected, arguing against an acquisition of naked DNA through a transformation process. Cell-free filtrates of the supernatant of the donor strain incubated with the recipient strain did not allow DNA transfer, suggesting the absence of transferable DNA released from the bacteria but protected from the action of a DNAse (inside phage particles or membrane vesicles). These results argue against a transfer of DNA by transformation or transduction and further point at conjugation as the most likely mechanism. However, since this conjugative process was observed in liquid medium under agitation, we wondered whether a strong shaking of the culture would disrupt the pilus-mediated interactions between bacterial cells, and therefore decrease the transfer frequency. Surprisingly, when we increased the agitation of the medium containing the donor and recipient cells to 130 rpm (which was vigorous under our experimental conditions), the frequency of transfer of the *irp2* locus was not affected (0.9×10^−8^). Electron microscopy analysis of IP32637 cells grown under conditions optimal for GDT4 did not reveal any pilus structures on the bacterial surface. In contrast, tightly aggregated bacilli that seemed to be connected by “bridges” were observed (Figure 4).

### IS–mediated DNA mobilization

We noted that after pUC4K transfer, the plasmid sizes of pUC4K in 10 different transconjugants were variable. This was confirmed after digestion of the 10 plasmids with *Nde*I, an enzyme that has a single restriction site in pUC4K. Three plasmids (rpUC4K-1 to -3) had the expected pUC4K size, while the seven others (rpUC4K-4 to -10) had a size superior to that of the original molecule (data not shown), indicating that various types of rearrangements had occurred during plasmid transfer. Remarkably, a search for the potential transposition of pGDT4-borne IS (IS*Yps1*, IS*L3*, IS*Yps2*, IS*NCY* or IS*Yps3*) on rpUC4K molecules by PCR (primers described in Table S1) showed that all seven larger size recombinant plasmids (rpUC4K-4 to -10) harbored IS*Yps1*.

**Figure 5:**
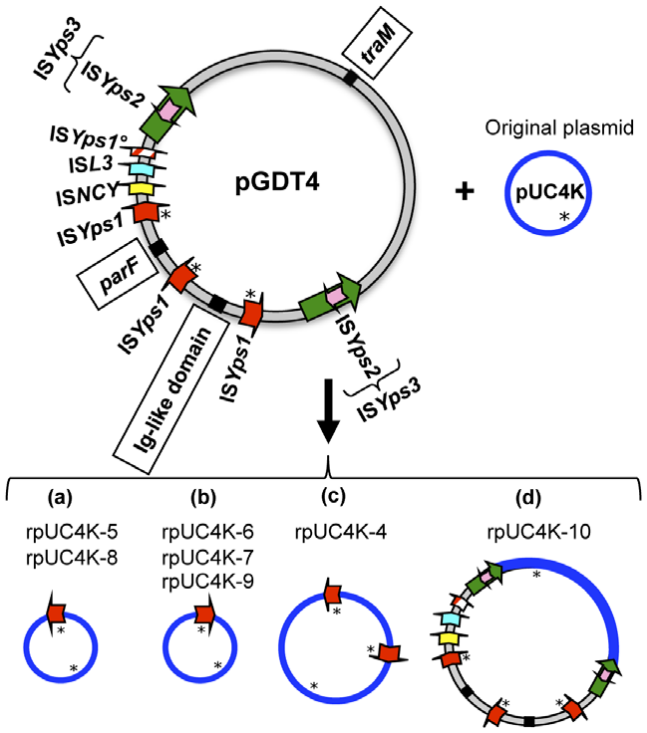
Schematic representation of seven recombinant pUC4K molecules recovered from transconjugants. Ten transconjugants resulting from the co incubation of 637(pUC4K) and 637-Nal^R^ were analyzed. The donor plasmid pGDT4 harbors two copies of IS*Yps1* in direct orientation and one copy in opposite direction, two copies of IS*Yps3* transposon including IS*Yps3* and IS*Yps2* transposases, one IS*L3* and one IS*NCY*. IS*Yps1*° is a truncated copy of IS*Yps1*. Stars indicate *Xho*I restriction sites. Genes used to search by PCR for the presence of inter-IS*Yps1* regions are boxed.

rpUC4K-5 to -9 had a size compatible with the acquisition of a single IS*Yps1* copy. Digestion with *Xho*I, an enzyme that cuts once in pUC4K and once in IS*Yps1*, yielded two restriction fragments, thus confirming the presence of a single IS*Yps1* copy. However, two distinct restriction profiles were observed, one for rpUC4K-5 and -8 and one for rpUC4K-6, -7 and -9 (data not shown), indicating the occurrence of different genetic rearrangements. Sequencing of the regions encompassing the IS*Yps1* insertion site in one recombinant plasmid of each group (rpUC4K-5 and rpUC4K-6) demonstrated that the IS was inserted at two different sites, approximately 100 bp apart, and in opposite orientation (Figure 5a and 5b). The insertion generated an 8 bp duplication of the target sequence: AAAATAGG in rpUC4K-5 and TATTTGAA in rpUC4K-6.

rpUC4K-4 had a size superior to that of the above five plasmids. *Xho*I digestion revealed the presence of two IS*Yps1* copies on this plasmid (Figure 5c). To determine whether the region located between these two IS*Yps1* copies on rpUC4K-4 corresponded to a portion of pGDT4, a PCR amplification of three pGDT4 genes (the Ig-like domain, *parF* and *traM*), each located between two different IS*Yps1* copies on pGDT4 (Figure 5) was performed. No positive signal was detected, suggesting that the region located between the two IS*Yps1* is a duplicated portion of pUC4K (Figure 5c).

The rpUC4K-10 plasmid was different from all others since the PCR analysis showed that it carries all five pGDT4-borne IS, as well as the Ig-like domain and *parF* genes (but not *traM*, Figure 5d). rpUC4K-10 has thus most likely acquired the entire pGDT4 sequence located between the two IS*Yps3* transposons (Figure 5).

Altogether, these results show that most pUC4K transfers generated a variety of genetic modifications that were systematically accompanied by the transposition of the pGDT4-borne IS*Yps1* element.

### Replicative transposition of IS*Yps1* and formation of cointegrates

IS*Yps1* belongs to the IS*6* family, known to transpose through replicative transposition. This mode of transposition gives rise exclusively to replicon fusions (cointegrates), in which the donor and target replicons are separated by two IS copies in direct orientation. The cointegrate can be subsequently resolved by recombination between the two IS copies [17]. To determine whether IS*Yps1* transposes through this mechanism, this IS was cloned into the suicide mobilizable vector pSW23T and introduced into a replication-permissive *E. coli* strain, yielding ω7249(pSW*Yps1.1*) (Table 1). After mating of this donor strain with ω4826, a non-replication permissive *recA-* recipient, ω4826::pSW*Yps1.1* transconjugants resulting from pSW*Yps1.1* integration into the recipient chromosome were obtained with a frequency of 8.5(±0.4)×10^−6^ (which corresponds to the frequencies of both conjugation and transposition, Table S5). Since the frequency of conjugation under these conditions was found to be 3.4(±0.9)×10^−3^ (Table S5), the transposition frequency of IS*Yps1* is thus approximately 2×10^−3^. The genomic DNA of eight independent ω4826::pSW*Yps1.1* colonies were digested with *Hin*dIII (which cuts once in pSW23T and not in IS*Yps1*), and hybridized with an IS*Yps1* probe. All eight clones harbored, as expected, two integrated copies of IS*Yps1* (Figure S2). Of note, all clones exhibited different hybridization profiles.

To further determine whether association between IS*Yps1* and a conjugative plasmid allows cointegrate transfer, the IS was cloned on a non-mobilizable vector (pSW23) and introduced into an *E. coli* strain carrying the conjugative plasmid R388 (Table 1). After mating of the resulting pi3(R388, pSW*Yps1.2*) donor strain with the ω4826 recipient that cannot sustain pSW*Yps1.2* replication, ω4826(R388::pSW*Yps1.2*) transconjugants were independently selected on Cm (pSW*Yps1.2* tag) and Tmp (R388 tag) MH agar plates. Cm^R^ clones were found at a frequency of 9(±3)×10^−5^ (Table S5) and were all Tmp^R^, while in the absence of an IS*Yps1* carried on the pSW23, no Cm^R^ transconjugants were obtained (Table S5). This further demonstrates that *Yps1* drives the formation of cointegrates that can be subsequently transferred by conjugation. Under these conditions, R388 was transferred at a frequency of 2.2(±0.7)×10^−1^ (Table S5), indicating that transfer of these cointegrates occurs at high frequencies (≈2×10^−5^). To further characterize these events, the plasmid profiles of five independent R388::pSW*Yps1.2* cointegrates were analyzed after restriction with *Mfe*I (10 sites in R388, one in pSW*Yps1.2*). All five pSW*Yps1.2* insertions were in different locations on R388 (data not shown). The two *Mfe*I junction fragments from one of these R388::pSW*Yps1.2* cointegrates were cloned into the *Eco*RI site of pUC18 and the precise cointegrate location was determined by sequencing. Transposition of pSW*Yps1.2* occurred in the orf5 cassette of the R388 integron [18] and led, as for the two pUC4K insertion events analyzed above, to an 8 bp duplication. The duplicated sequence (GATCCGAG) was different from the other two, further indicating the absence of a specific integration site.

Our results thus demonstrate that IS*Yps1* is able to transpose into a variety of insertion sites by replicative transposition through cointegrate formation, mediating the transfer of potentially any piece of non-mobilizable DNA molecule.

## Discussion

We have evidenced a mechanism of HGT that convey the conjugative transfer or virtually any piece of chromosomal or plasmid DNA in a natural isolate of *Y. pseudotuberculosis*. This mechanism shares some characteristics with those previously described, but has several novel and unique properties.

GDT4 is not observed at temperatures ≥20°C and its efficiency increases as the temperature decreases. Although some conjugative plasmids have been previously shown to be self-transferable at 14°C but not at 37°C [19], [20], to our knowledge no plasmid able to conjugate at 4°C has ever been described. Temperature-dependent plasmid transfer is primarily mediated by H-NS and Hha proteins, which can be both plasmid and/or chromosome encoded [20]. The pGDT4 sequence did not reveal any gene encoding such proteins, but it is known that chromosomally encoded Hha and YmoA (equivalent to H–NS) act as thermoregulators in *Y. enterocolitica* [21]. These proteins (also encoded by the *Y. pseudotuberculosis* genome [8]) may thus modulate pGDT4 transfer at cold temperatures. Interestingly, H-NS is an integral part of bacterial stress response pathways and its function is known to be sensitive to changes in environmental conditions such as temperature [22], [23]. A cold stress could thus be a signal for the bacteria to transfer their genetic material by GDT4. The low temperature and an iron-poor liquid environment may also induce changes in the bacterial membrane structure, as observed for the closely related organism *Y. pestis*, in which the transcription patterns of various genes encoding components of the bacterial membrane were modified during iron starvation [24] or growth at 10°C [25]. These modifications might facilitate the formation of pores through which chromosomal DNA could translocate. Indeed, large cell aggregates in which bacteria appeared to be connected by bridges were observed. Similar tight bacterial contacts, designated conjugative junctions [26] or conjugational junctions [27] have been observed during RP4 or F-mediated mating of *E. coli*, respectively. However, these physical properties do not seem to be pGDT4-mediated, as we also observed large bacterial aggregates and possibly intercellular channels with IP32953, a strain that does not harbor this plasmid (data not shown). These bacterial aggregates have some similarities with biofilms in which bacteria are also closely connected. Biofilm formation occurs under natural conditions in a variety of bacterial species [28], including *Y. pseudotuberculosis* [29]. It could thus be hypothesized that GDT4 may take place between bacteria residing within biofilms in their natural ecological niches. This observation also suggests that acquisition of new functions, including virulence factors, by *Y. pseudotuberculosis* takes place in the environment rather than in a mammalian host.

Another characteristic feature of GDT4 is that transfer of chromosomal DNA occurs only in a liquid medium. Like other conjugative processes, GDT4 requires a pilus-like mating system and a mating channel to occur, as demonstrated by the fact that inactivation of the pGDT4-borne pilus apparatus abolished this mechanism. While some plasmids transfer better on plates, some others encoding long flexible pili allow DNA transfer efficiencies of the same magnitude in liquid and on solid media [30], [31], and this applied to pGDT4. In contrast, the absence of transfer of chromosomal DNA on agar was unexpected. Also unexpected was the fact that the efficiency of transfer in broth was not affected by a strong agitation, as opposed to recent findings showing that a vigorous shaking negatively affected the transfer of several conjugative plasmids, including the F' plasmid that encodes long and flexible pili [32]. Actually, growth in a liquid medium at low temperature could create the conditions optimal for GDT4. Indeed, this environment might be more favorable for the formation of tight bacterial aggregates and inter-cellular bridges through which long stretches of chromosomal DNA could transit.

pGDT4 also triggered the conjugative transfer of the non-mobilizable plasmid pUC4K. Similarly, transfer of the non mobilizable plasmid pBR325 by an RP4::miniMu mobilizing plasmid was previously observed [33], but the mechanism underlying this genetic transfer was not characterized. *cis*-mobilization of non-mobilizable plasmid DNA can occur after integration of a conjugative plasmid into the genetic element to be transferred. Integration arises either by homologous recombination between identical elements, often two copies of the same IS located on each DNA molecule (as for the Hfr formation in *E. coli* [34]), or through the formation of cointegrates mediated by specific transposons or ISs [35]. Integration of pGDT4 into pUC4K could not occur via homologous recombination as the two replicons do not share any common IS or identical DNA sequences. However, pGDT4 carries IS*Yps1*, an IS which is predicted to belong to the IS*6* family (http://www-is.biotoul.fr/is.html). IS*Yps1* is the second IS of the IS*6*-type identified in the genus *Yersinia* [36]. Members of this family have the capacity to create cointegrates by replicon fusion in the absence of a homologous IS on the target DNA [37]. Furthermore Tn*3*, which also moves by replicative transposition, has been found to mediate cis-mobilization of non mobilizable plasmids by this mechanism [35]. The fact that several rpUC4K plasmids obtained after pGDT4-mediated transfer carried a copy of IS*Yps1* argues for a role of this IS in pGDT4 integration into its target. We have demonstrated that IS*Yps1* is indeed transposing through replicative transposition. IS*Yps1* has a low specificity of recognition of the target sequence, as attested by our observation that the three insertion sites sequenced (two on pUC4K and one on R388) were different. This model also predicts the formation of cointegrates carrying two copies of the IS element, each flanking the sequence of the donor plasmid. We do have observed the formation of cointegrates between R388 and pSW*Yps1* flanked by the expected IS*Yps1* copies. A resolution step, which occurs through homologous recombination between the two IS copies, is then required to separate the donor and target replicons, leaving a single IS copy in the target and restoring the donor plasmid. The rpUC4K-5 to -9 molecules that were found to carry one IS*Yps1* copy are most likely the results of such a resolution event. The presence on one recombinant plasmid (rpUC4K-10) of a portion of pGDT4 carrying IS*Yps1*, IS*Yps2*, IS*Yps3*, IS*NCY* and IS*L3* indicates that additional, complex rearrangements involving the Tn*3*-like transposon IS*Yps3* can also occur. Finally, the existence in some transconjugants of pUC4K plasmids with a size identical to that of the original molecule could be the result of the resolution of cointegrates containing pUC4K concatemers.

**Figure 6:**
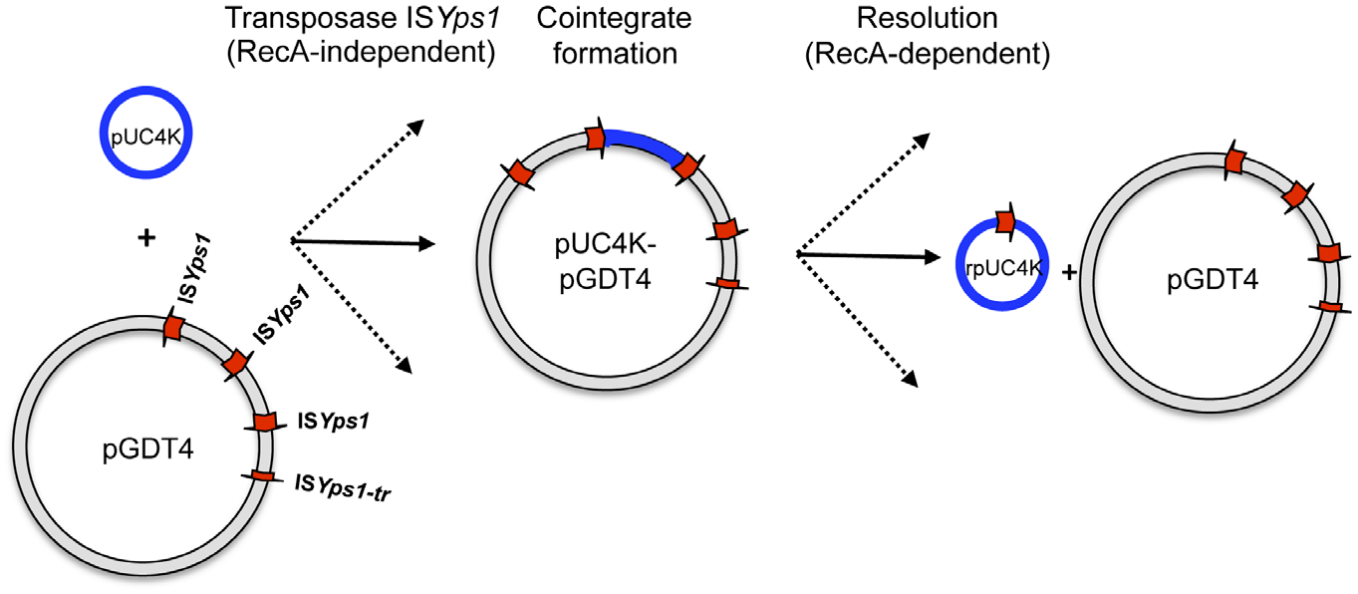
Model proposed for pUC4K mobilization based on the transposition mechanism of the IS*6* family. Transposase-mediated replicon fusion of the two plasmid molecules generates a cointegrate carrying an additional copy of IS*Yps1* in the same orientation. Although only one type of cointegrate is represented here, different types of cointegrates mediated by each IS*Yps1* copy can be generated (indicated by dashed arrows). RecA-dependent homologous recombination between any two copies of IS*Yps1* present on the cointegrate will either regenerate the donor plasmid, leaving a single IS copy in the target pUC4K or create a rpUC4K containing a portion of pGDT4. Figure adapted from Mahillon J. and Chandler M. [17].

GDT4 thus represents a remarkable illustration and validation of the model of Tn*3*-mediated transmission of non conjugative plasmids proposed by Crisona *et al.* in the 1980's [35]. Following this model, the first step is the integration of pGDT4 into its target DNA by IS*Yps1*-mediated replicon fusion during plasmid replication (Figure 6). As mentioned above, this generates a cointegrate which carries the two replicons separated on each side by an IS*Yps1* copy. This cointegrate then uses the conjugative machinery encoded by pGDT4 to promote its transfer to the recipient strain. The final step is the resolution of the cointegrate by homologous recombination between two IS*Yps1* copies or any other duplicated sequence present in the cointegrate. Since pGDT4 carries several IS*Yps1*, the resolved molecules have different sizes and DNA composition.

GDT4 is also able to mediate the translocation of chromosomal DNA, most likely by integration into the bacterial chromosome and transfer in a Hfr-like manner. The Hfr mechanism is one of the earliest and best described examples of chromosomal transfer and is mediated by the F plasmid of *E. coli* [38]–[40]. F integrates stably into the *E. coli* chromosome through homologous recombination between IS copies present on both the F plasmid and the bacterial chromosome [38]–[42] to create Hfr strains, with transfer origins located at different chromosomal loci [43], [44]. In contrast to the classical Hfr mechanism, integration of pGDT4 into the chromosome probably occurs, as in pUC4K, via the IS*Yps1*-mediated replication fusion mechanism. At least three pieces of evidence support this hypothesis: (i) no IS element is shared by pGDT4 and the IP32637 chromosome, in contrast to what is expected for the Hfr mechanism, (ii) in *Y. pseudotuberculosis*, three distantly located chromosomal loci (*irp2^K^*, *ureB^S^* and *or5076^T^*) were transferred with similar frequencies, and (iii) in all eight *E. coli* transconjugants analyzed, pSW*Yps1* was inserted at different sites on the chromosome. IS*Yps1* thus appears to have a very low specificity of recognition, allowing its insertion at multiple sites on bacterial plasmids and chromosomes. After mobilization of the chromosomal fragment adjacent to the pGDT4 integration site and transfer to a recipient strain, following the Hfr-type transfer model, homologous recombination between the incoming DNA and the chromosome is expected to take place, leaving no trace of pGDT4 in the chromosome of the transconjugant. Our previous observation that RecA activity is necessary in the recipient, but not in the donor strain for chromosomal transfer [3], and the results of the present study showing that pGDT4 is absent from some transconjugants that have acquired chromosomal genes further support this model of horizontal transfer. Our study thus validates the model proposed by Willets *et al.* in the 1980's for the mobilization of the *E. coli* chromosome via the formation of a cointegrate with the R68.45 plasmid during IS*21* transposition [45]. Such cointegrate formations were widely used at that time to establish the genetic map of various bacterial species (see for instance [46], [47]). Most importantly our results show, without the need for heterologous plasmids like RP4 or R68.45, that this type of chromosomal conjugative transfer may occur under natural conditions in wild type bacterial pathogens carrying endogenous plasmids.

The capacity of wild type bacteria to naturally transfer large pieces of chromosomal DNA following the typical Hfr mechanism of homologous recombination between identical IS copies on the chromosome and the plasmids has been documented in a variety of bacteria, including extremophiles [48], Gram-positive cocci [49], and actinomycetes [50]. What we describe here is certainly a less constrained and more powerful mechanism, as it only requires the presence of an IS of the IS*6* family on a conjugative replicon to generate cointegrates able to drive the horizontal transfer of any piece of DNA (chromosomal or episomal). It is remarkable that a high density of IS is commonly observed on plasmids. For instance the *Shigella* plasmid pWR100 carries 93 copies of complete or truncated IS belonging to 21 different types [51]. Thus, more than being IS depository, this location may reflect the broad selective advantage brought by plasmid/IS associations as a chromosomal transfer device. Such a ‘genetic symbiosis’, offers a means for the natural transfer of large blocks of genes conferring new metabolic properties or virulence functions. According to our model, GDT4 does not leave any signature in the recipient genome in most instances, and therefore its contribution to the numerous horizontal gene exchanges that shape bacterial genomes can hardly be quantified. However, according to the ISfinder database, approximately 5% of the known IS belong to the IS*6* and Tn*3* families, which use a replicative transposition mechanism. As they are found in all bacterial and archaeal phyla, the mechanism we describe here might be responsible for a substantial fraction of gene exchanges occurring among bacterial species.

Remarkably, this mechanism of DNA transfer was optimal when the bacteria were grown under conditions (low temperatures, iron poor medium, biofilm-like bacterial aggregates) that might be close to those met by these microorganisms in their normal ecological niches. This natural GDT mechanism may thus play a significant role in bacterial evolution, genetic polymorphism, pathogenesis and adaptation to new environmental conditions.

## Methods

### Bacterial strains and growth conditions

Bacterial strains used in this study are listed in Table 1 and Table S1. Wild type strains were taken from the collection of the Yersinia Research Unit (Institut Pasteur). Bacteria were grown in LB (Luria Bertani) or MH (Mueller Hinton) medium for 24 h at 28°C (*Yersinia*) or 37°C (*E. coli*) with agitation, or for 48 h on LB or MH agar plates. When necessary, kanamycin (Kan: 100 µg ml^−1^), rifampicin (Rif: 100 µg ml^−1^), nalidixic acid (Nal: 25 µg ml^−1^), spectinomycin (Spe: 50 µg ml^−1^), tetracycline (Tc: 15 µg ml^−1^), chloramphenicol (Cm: 25 µg ml^−1^), trimethoprim (Tmp: 20 µg ml^−1^), thymidine (dT: 0.3 mM) or the iron chelator αα'-dipyridyl (0.2 mM, Sigma) were added to the medium.

### Mutagenesis of chromosomal or plasmid genes

Spe (*aadA*) or Tmp (*dfr*) non-polar cassettes were PCR-amplified using primers described in Table S1, and pSW25 [52] or pGP704N-*dfr* [3] as templates, respectively. All allelic exchanges of chromosomal or plasmid genes by an antibiotic resistance cassette were done following the LFHR-PCR procedure [53]. The Spe and Tmp cassettes were introduced into the chromosomal *ureB* and *or5076* genes, respectively, using primers that amplify upstream and downstream fragments of *ureB* and *or5076*, as shown on Figure S1 and Table S1. To label pGDT4, the plasmid was digested with *Hin*dIII and a 4 kb fragment (pGDT4-4kb) was purified and cloned into pUC18. Approximately 600 bp of each extremity of the cloned fragment were sequenced. These sequences were then used to design primers (358A/B and 359A/B, Table S1) that served for allelic exchange between the Tmp cassette and the target region of pGDT4 in strain 637-*irp2^K^-ureB^S^*. Correct insertion of the Tmp cassette was confirmed by PCR using primer pair 358A/359B. Mutagenesis of the *pil* region was done by replacing the pGDT4 region extending from *pilL* (pGDT4_0086) to *pilV* (pGDT4_0097) by a Tmp cassette, using primer pairs 773A/B and 774A/B (Table S1). The various antibiotic-tagged derivatives cured of pKOBEG-*sacB* were selected on sucrose plates.

### Growth conditions for DNA transfer and determination of the transfer frequency

Optimal conditions for chromosomal DNA transfer in *Y. pseudotuberculosis* have been previously described [3]. Briefly, the donor strain (usually Rif^R^) harboring chromosomal loci labeled with antibiotic cassettes and the recipient strain (usually Nal^R^) were grown overnight in LB at 28°C with agitation. Equal amounts (5×10^6^) of donor and recipient cells were mixed in 25 ml of LB-αα' and grown at 4°C with mild rotary agitation (80 rpm) for 4 days. Donor and recipient bacteria were quantified on Rif and Nal plates, respectively, and transconjugants were selected on Nal plates containing the appropriate antibiotic. To ensure that the colonies were not spontaneous Nal^R^ mutants of the Rif^R^ recipient strain, the Rif susceptibility of the transconjugants was systematically checked. For every single DNA transfer experiment, 10 to 20 transconjugant colonies were analyzed by PCR for the acquisition of the corresponding antibiotic-tagged locus with primer pairs 233B/166, 92A/322B and 348B/346A (Table S1) as indicated on Figure S1. When the transfer of the *irp2^K^* locus was analyzed, the acquisition of the entire HPI by the recipient strain was further checked with primer pairs A10/144A and A9/143B (Figure S1 and Table S1). The frequency of DNA transfer was determined as the number of Nal^R^ (or Kan^R^, Spe^R^, Tmp^R^) Rif^S^ transconjugants per Rif^R^ donor cells. To determine whether free DNA molecules in the medium could mediate GDT4, the donor bacteria 637-*irp2^K^-ureB^S^* and the recipient 637ΔHPI-Nal^R^ were co-incubated in the presence of 100 U/ml of DNAse in the culture medium. The activity of the DNAse under these conditions was checked by adding 1 ug/ml of bacterial DNA to the culture medium and by observing that the added DNA was degraded.

### Transfer of pGDT4^T^

Transfer of pGDT4^T^ was studied after incubation of the donor (637-*irp2^K^-ureB^S^*(pGDT4^T^)) and various Nal^R^ recipient cells for four days at 4, 28 or 37°C in liquid or solid media. On solid medium, 2×10^8^ donor and recipient cells were mixed on a 0.45 µm nitrocellulose filter (Millipore) and at the end of the incubation period, the bacterial mixture was suspended in 1 ml of MH. Donor and recipient cells were quantified on MH-Rif and MH-Nal plates, respectively. Transconjugants having acquired pGDT4^T^ were identified as Nal^R^/Tmp^R^/Rif^S^ colonies. In each transfer experiment, 10 transconjugants were analyzed by PCR for the presence of pGDT4^T^ with primer pair 358A/346B (Table S1). Finally, the pGDT4^T^ transfer frequency was calculated as the number of Nal^R^/Tmp^R^/Rif^S^ transconjugants per donor cells.

### Sequencing of pGDT4

One transconjugant resulting from the co-incubation of the 637-*irp2^K^-ureB^S^* donor strain with the 637c-Nal^R^ recipient was used to obtain a plasmid extract which contained only pGDT4. Sequencing was performed using the whole genome shotgun strategy [54]. A 2–3 kb insert library was generated by random mechanical shearing of pGDT4 DNA and cloning into pcDNA-2.1 (Invitrogen). Recombinant plasmids were used as templates for cycle sequencing reactions consisting in 35 cycles (96°C for 30 s; 50°C for 15 s; 60°C for 4 min) in a thermocycler, using the Big dye terminator kit (V3.1, Applied Biosystems). Samples were precipitated and loaded onto a 96-lane capillary automatic 3700 DNA sequencer (Applied Biosystems). In an initial step, 1000 sequences from the library were assembled into 5 contigs using the Phred/Phrap/Consed software [55], [56] (8-fold sequence coverage). Consed was used to predict links between contigs. PCR products amplified from the pGDT4 template were used to fill gaps and to re-sequence low quality regions using primers designed by Consed. Physical gaps were closed using combinatorial PCR. The correctness of the assembly was confirmed by ensuring that the deduced restriction map was identical to the one obtained experimentally. The *traX* and *traY* genes fusion into a single *traXY* gene was checked by re-sequencing this locus on the original pGDT4 DNA preparation.

IS*Yps1, ISYps2 and ISYps*3 designation were attributed by the ISfinder database (http://www-is.biotoul.fr/). The nucleotide sequence of pGDT4 has been submitted to the EMBL database under accession number FM178282. Details and properties of the different IS*Yps* characterized in this work are accessible through the ISFinder web site.

### Electron microscopy

Bacteria were negatively stained with 2% uranyl acetate onto glow discharged copper grids. The samples were observed in a Jeol 1200EXII and/or a JEM 1010 (Jeol) equipped with a Keenview camera (Eloise) at 80-kV accelerating voltage. Images were recorded with an Analysis Pro Software version 3.1 (Eloise).

### IS*Yps1* transposition assay

The sequence corresponding to the IS*Yps1* copy carried on rpUC4K-6, flanked by its 113 bp upstream and 138 bp downstream regions, was amplified using primers 1039/1040 and cloned as an *Eco*RI-*Bam*HI insert into the suicide mobilizable vector pSW23T [52], giving rise to pSW*Yps1.1*. This plasmid was then introduced into *E. coli* ω7249 [57], a strain allowing pSW23T replication and conjugative transfer. Conjugation between this donor strain and *E. coli* ω4826 was performed as previously described [57], The frequency of conjugation–transposition frequency was calculated as the number of Cm^R^ transconjugants (ω4826::pSW*Yps1.1*) per total number of recipients (Tc^R^). The conjugation frequency was established in parallel by conjugation from the same donor ω7249(pSW*Yps1.1*) to a ω4826 pir+ derivative (obtained through transformation with plasmid pSU38Δpir which expresses *pir* [52]). The frequency of illegitimate recombination of the pSW23T which can lead to Cm^R^ transconjugants was established by conjugation between donor ω7249(pSW23T) and ω4826, and found to be 4.6(±1.7)×10^−8^. Genomic DNA from 8 independent ω4826::pSW*Yps1.1* colonies were extracted using QIAGEN Genomic Tips and buffer set, digested with *Hin*dIII, and hybridized with a probe internal to IS*Yps1* (generated by PCR amplification with primers 1041/1044 and labeled with α-32P dCTP, using the Random Primed labeling kit (Roche)).

### R388::pSW*Yps1.2* cointegrate formation assay

The *Eco*RI-*Bam*HI fragment carrying IS*Yps1* was transferred from pSW*Yps1.1* to the non-mobilizable version of pSW23 [52], giving rise to pSW*Yps1.2*. This plasmid was then introduced into the *E. coli* pi3 *pir*+ strain that harbors the IncW conjugative plasmid R388, which does not carry any IS (GenBank BR000038), giving rise to pi3(R388, pSW*Yps1.2*). Conjugation of this donor strain with ω4826 yielded ω4826(R388::pSW*Yps1.2*). The frequency of cointegrate formation after mating was calculated as the number of Cm^R^ transconjugants per total number of recipients harboring R388 (Tmp^R^). The ability of R388 to form transferable cointegrates with pSW23 in the absence of IS*Yps1* was assessed in the same conditions by replacing pSW*Yps1.2* by pSW23 in the pi3(R388) donor, and found to be inferior to 10^−9^.

## Supporting Information

Figure S1 Genetic map and location of primers used to insert antibiotic cassettes in the chromosomal *irp2*, *ureB* and *or5076* genes. *kan*: kanamycin cassette, *spe*: spectinomycin cassette, *tmp*: trimethoprim cassette.
(PDF)

Figure S2 IS*Yps1* probing of 8 w4826::pSW*Yps1.1* transconjugants after suicide conjugation of pSW*Yps1.1*.
(PDF)

Table S1 Primers used in this study. The location of several of these primers is illustrated on Figure S1. Lower case letters indicate portions of *spe* (310B, 311A) or *tmp* (347B, 348A, 358B, 359A, 773B and 774A) sequences.
(PDF)

Table S2 *Y. pseudotuberculosis* and *Y. pestis* strains analyzed for the presence of pGDT4. These strains were taken from the collection of the Yersinia Research Unit (Institut Pasteur). +: amplification of a fragment of the expected size; −: no amplification of a fragment of the expected size. A: Antiqua, M: Medievalis, O: Orientalis.
(PDF)

Table S3 Frequencies of GDT4-mediated transfer under various conditions. The conditions of co-incubation of the donor and recipients strains and the number of experiments are described in the legend of Figure 2.
(PDF)

Table S4 Annotation of the putative open reading frames of pGDT4.
(PDF)

Table S5 IS*Yps1* transposition and cointegrate formation frequencies.
(PDF)
