# Supplementary figures and images for: A Natural System of Chromosome Transfer in Yersinia pseudotuberculosis
Source: PLoS Genet. 2012 Mar 8;8(3):e1002529. doi: 10.1371/journal.pgen.1002529 (PMC3297565; doi:10.1371/journal.pgen.1002529)

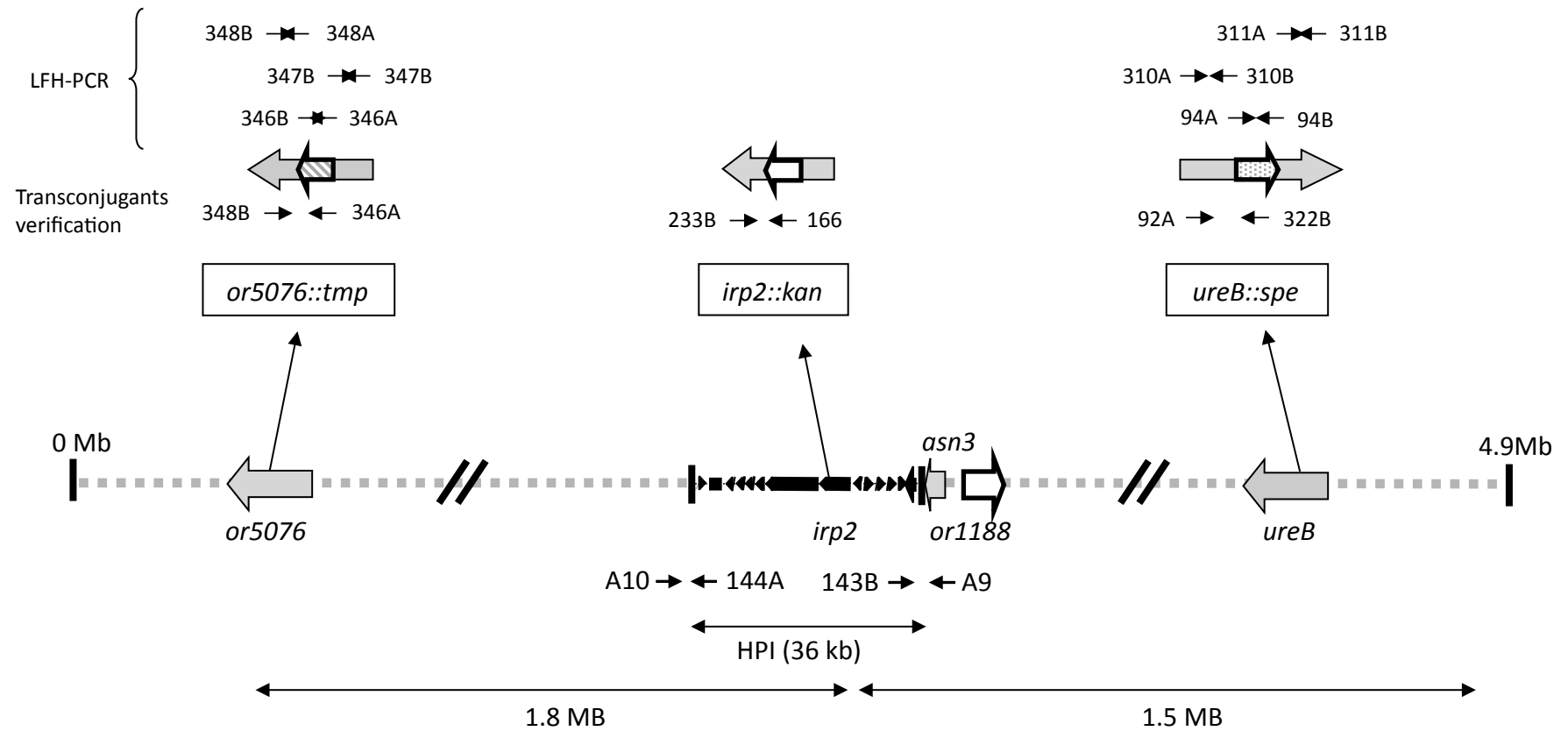

Supplement: Figure S1 — Genetic map and location of primers used to insert antibiotic cassettes in the chromosomal irp2, ureB and or5076 genes. kan: kanamycin cassette, spe: spectinomycin cassette, tmp: trimethoprim cassette. (PDF) [file pgen.1002529.s001.pdf]

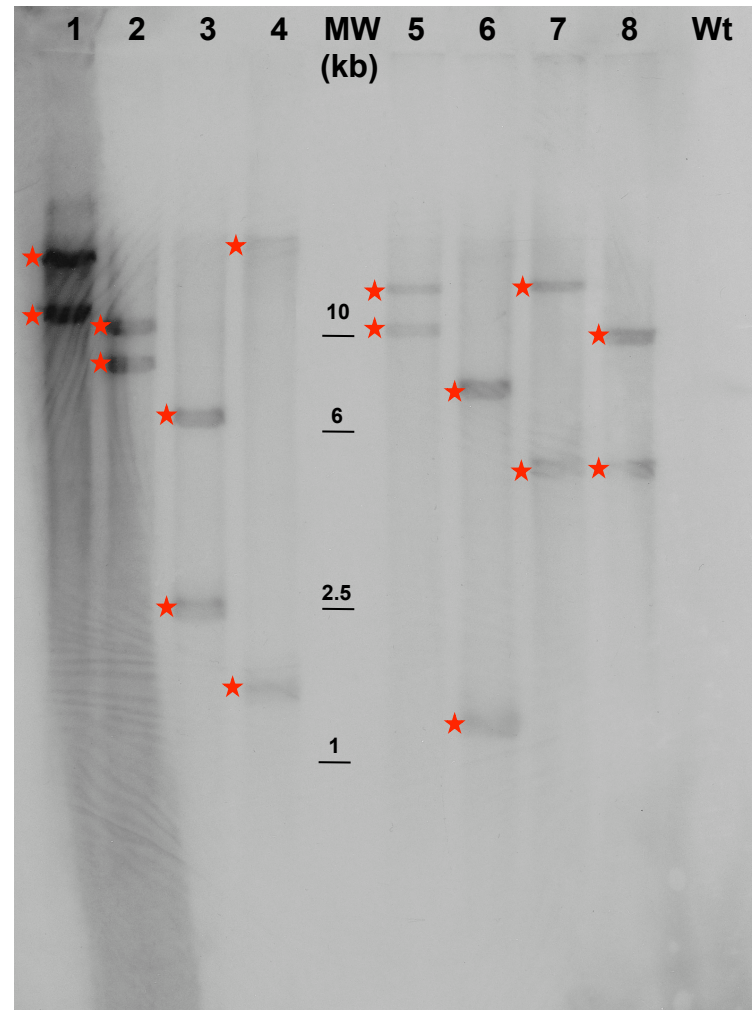

Supplement: Figure S2 — ISYps1 probing of 8 w4826::pSWYps1.1 transconjugants after suicide conjugation of pSWYps1.1. (PDF) [file pgen.1002529.s002.pdf]
