# Supplementary material for: A Natural System of Chromosome Transfer in Yersinia pseudotuberculosis
Source: PLoS Genet. 2012 Mar 8;8(3):e1002529. doi: 10.1371/journal.pgen.1002529 (PMC3297565; doi:10.1371/journal.pgen.1002529)
Supplement: Table S1 — Primers used in this study. The location of several of these primers is illustrated on Figure S1. Lower case letters indicate portions of spe (310B, 311A) or tmp (347B, 348A, 358B, 359A, 773B and 774A) sequences. (PDF) [file pgen.1002529.s003.pdf]

| Primers | Sequence | Locus | Location |
|---|---|---|---|
| 210A | 5’-TATGGGTATAAATGGGCTCGG-3’ | *kan* | pUC4K |
| 210B | 5’-CACCGAGGCAGTTCCATAGG-3’ | *kan* | pUC4K |
| 166 | 5’-TCCTATGGAACTGCCTCGGT-3’ | *kan* | pUC4K (internal to *kan*) |
| 294A | 5’-CACTGGCATTTAATAACGCGTC-3’ | *spe* | pSW25 |
| 294B | 5’-TTAATCACTTTACTTTTATC-3’ | *spe* | pSW25 |
| 322B | 5’-GCTGGCAAGCGTGCCATTGCCC-3’ | *spe* | pSW25 (internal to *spe*) |
| 346A | 5’-ATATCTGAGCTGTTGACAATTAATCATCC-3’ | *tmp* | pGP704-*dfr* |
| 346B | 5’-CCACCAAACTTAGTTGATGCGTTCAAGCG-3’ | *tmp* | pGP704-*dfr* |
| 233B | 5’-GTCAAAAATTGCTGCTCGCTACGCA-3’ | *irp2* | Chromosome |
| 143B | 5’-AAGGGACATATGGGGGTCAC-3’ | HPI right border | Chromosome |
| 144A | 5’-ATTTGTAGTGCCGTAGGA-3’ | HPI right border | Chromosome |
| A9 | 5’-ACTAGACGTAGATAAGCGAG-3’ | HPI left border | Chromosome |
| A10 | 5’-AAACCTTGGGCTGGCGCTAC-3’ | HPI left border | Chromosome |
| 310A | 5’-TATCATACAAATAGGGTGGTGGATAATTTG-3’ | *ureB* | Chromosome |
| 310B | 5’-gtatttattcggcgcaattgCATCAGTCACAGTAATGATAGAA-3’ | *ureB* | Chromosome |
| 311A | 5’-taaactgcttggtgccagccaatgaTAACGCCTTGTTTACAGAGC-3’ | *ureB* | Chromosome |
| 311B | 5’-GTTGGACAGCGCATGATAGGCCTG -3’ | *ureB* | Chromosome |
| 92A | 5’-TGAGCGCGGTAAAAATACTGAGCG-3’ | *ureB* | Chromosome |
| 347A | 5’-GGACCATGTGATCATTGCCGAGGACC -3’ | *or5076* | Chromosome |
| 347B | 5’-ggatgattaattgtcaacagctcATCACCATCGACGCGCCTAGCC-3’ | *or5076* | Chromosome |
| 348A | 5’-cggcgcttgaacgcatcaactaatGCCTTTAATGCTTATCGGGGAC-3’ | *or5076* | Chromosome |
| 348B | 5’-CTTTATGTATATTGCAATGATTAGCACAGG-3’ | *or5076* | Chromosome |
| 358A | 5’-CAGGGCTGCAGCCCTGACGGTCTACAACC-3’ | pGDT4.*seq* (pGDT4-0029) | pGDT4 |
| 358B | 5’-ggatgattaattgtcaacagctc TTGACGGAATACGTTCCCAGTC-3’ | pGDT4.*seq* (pGDT4-0029) | pGDT4 |
| 359A | 5’-cggcgcttgaacgcatcaactaatTCACCCGTGTTTTATATAACCG-3’ | pGDT4.*seq* (pGDT4-0029) | pGDT4 |
| 359B | 5’- ATGTCATATGTGCAATGCTGCACACCAG-3’ | pGDT4.*seq* (pGDT4-0029) | pGDT4 |
| 723A | 5’-TCCCATATCGACCCTTCAGC-3’ | Ig-like domain | pGDT4 |
| 723B | 5’-GCACTGGGATATTGTTGTCGTC -3’ | Ig-like domain | pGDT4 |
| 724A | 5’-AACTTGTCTTGTGCGCTCTG -3’ | *parF* | pGDT4 |
| 724B | 5’-CTTTGGCCGCACTGTCTTTC -3’ | *parF* | pGDT4 |
| 725A | 5’- CACCTTATCAAGCCGCATTCC -3’ | *traM* | pGDT4 |
| 725B | 5’- CTGAGAAGATGAATCGCTGTGC-3’ | *traM* | pGDT4 |
| 727A | 5’-GTTCTGTCGCATTAAGGCGTTA-3’ | IS*Yps1* | pGDT4 |
| 727B | 5’-GGTTTCATCCATGCGCCACCGA-3’ | IS*Yps1* | pGDT4 |
| 729A | 5’-AGAAACCGCTCGCGACTTTCTG-3’ | IS*NCY* | pGDT4 |
| 729B | 5’-GATAAGGATAAGGTATCACCTGGCC-3’ | IS*NCY* | pGDT4 |
| 730A | 5’-GTGAGATGCTGTTTAAGACCTG-3’ | IS*Yps2* | pGDT4 |
| 730B | 5’-GAATCTTCTGACAACGGTAATGCA-3’ | IS*Yps2* | pGDT4 |
| 731B | 5’-CTGTCCATCAAATACCGCAAGG-3’ | IS*L3* | pGDT4 |
| 731A | 5’-ATGATGGGCTATACCCCCGAAC-3’ | IS*L3* | pGDT4 |
| 732A | 5’-GAACCCGAGGCAATATTGAGGCGA-3’ | IS*Yps3* | pGDT4 |
| 732B | 5’-CGGTCATTCGCTGACGCCAGGTC-3’ | IS*Yps3* | pGDT4 |
| 773A | 5’-CACACATCAATGCTGCTCCCTATTATATTGGGTGGCTGCAGCC-3’ | *pilL* | pGDT4 |
| 773B | 5’-cggcgcttgaacgcatcaactaaACGGAATACGCCATTCAATGC-3’ | *pilL* | pGDT4 |
| 774A | 5’-ggatgattaattgtcaacagctcTTAGCGTTCATCAGGGTG-3’ | *pilV* | pGDT4 |
| 774B | 5’-CTGCATCGCGAGTTAATGTCCCGATTTCACACGCCTGACC-3’ | *pilV* | pGDT4 |
| 1039 | 5'-GCCCGAATTCCAAAAAAGGGAATAAGGGCGACACG-3' | IS*Yps1* | rpUC4K-6 |
| 1040 | 5'- CGGGGGATCCGACGAAAGGGCCTCGTGATACG-3' | IS*Yps1* | rpUC4K-6 |
