# Supplementary material for: A Natural System of Chromosome Transfer in Yersinia pseudotuberculosis
Source: PLoS Genet. 2012 Mar 8;8(3):e1002529. doi: 10.1371/journal.pgen.1002529 (PMC3297565; doi:10.1371/journal.pgen.1002529)
Supplement: Table S2 — Y. pseudotuberculosis and Y. pestis strains analyzed for the presence of pGDT4. These strains were taken from the collection of the Yersinia Research Unit (Institut Pasteur). +: amplification of a fragment of the expected size; −: no amplification of a fragment of the expected size. A: Antiqua, M: Medievalis, O: Orientalis. (PDF) [file pgen.1002529.s004.pdf]

Strain	Country	Serotype or Biotype	pGDT4.*seq* detection by PCR
Y. pseudotuberculosis			
IP33038	Australia	I	-
IP30437	Canada	I	-
IP32950	France	I	-
IP32953	France	I	-
IP32954	France	I	-
IP33005	Germany	I	-
IP30284	Italy	I	-
IP32790	Italy	I	-
IP32533	New Zealand	I	-
IP32939	Romania	I	-
IP33134	Russia	I	-
IP30642	Tunisia	I	-
IP31878	Tunisia	I	-
IP32665	Yugoslavia	I	-
IP32581	Belgium	II	-
IP30215	Denmark	II	**+**
IP30911	Holland	II	-
IP32589	New Zealand	II	-
IP32584	Spain	II	-
IP33023	Switzerland	II	-
IP32938	Argentina	III	-
IP32992	Australia	III	-
IP33108	Bulgaria	III	-
IP33051	France	III	-
IP32802	Italy	III	-
IP32544	South Africa	III	-
IP32889	Spain	III	-
IP32984	Spain	III	-
IP31411	Denmark	IV	-
IP31833	England	IV	-
IP32687	France	IV	-
IP30151	Sweden	IV	-
IP31830	UK	IV	-
IP32699	France	V	**+**
IP32821	France	V	-
IP32952	France	V	-
IP33061	Germany	V	-
IP32816	Japan	V	-
IP32463	Switzerland	V	-

Y. pestis			
IP554	Kenya	A	-
IP544	Kenya	A	-
IP549	Belgian Congo	A	-
IP677	Kenya	A	-
IP538	Kenya	A	-
IP540	Kenya	A	-
IP542	Kenya	A	-
IP545	Kenya	A	-
IP547	Kenya	A	-
IP550	Kenya	A	-
IP551	Kenya	A	-
IP553	Kenya	A	-
IP552	Kenya	A	-
IP548	Belgian Congo	A	-
IP541	USSR	A	-
IP611	Japan	A	-
IP546	Nepal	A	-
IP565	Turkey	M	-
IP556	Kurdistan	M	-
IP560	Kurdistan	M	-
IP617	Iran	M	-
IP669	Kurdistan	M	-
IP670	Kurdistan	M	-
IP1594	Kurdistan	M	-
IP557	Kurdistan	M	-
IP516	Kurdistan	M	-
IP519	Kurdistan	M	-
IP616	Iran	M	-
IP241	Madagascar	O	-
IP507	Vietnam	O	-
IP524	Senegal	O	-
IP528	Madagascar	O	-
IP567	Brazil	O	-
IP568	Brazil	O	-
IP613	Burma	O	-
IP643	Madagascar	O	-
IP695	Germany	O	-
IP696	Germany	O	-
IP772	Vietnam	O	-
IP820	Vietnam	O	-
IP940	Vietnam	O	-
IP989	Vietnam	O	-
IP1537	Namibia	O	-
IP1540	Namibia	O	-
IP1541	Namibia	O	-
IP608	Israel	O	-
CO92	USA	O	-
IP1220	Vietnam	O	-
IP304	Madagascar	O	-
IP571	Brazil	O	-
IP685	Germany	O	-
