# Supplementary material for: A Natural System of Chromosome Transfer in Yersinia pseudotuberculosis
Source: PLoS Genet. 2012 Mar 8;8(3):e1002529. doi: 10.1371/journal.pgen.1002529 (PMC3297565; doi:10.1371/journal.pgen.1002529)
Supplement: Table S3 — Frequencies of GDT4-mediated transfer under various conditions. The conditions of co-incubation of the donor and recipients strains and the number of experiments are described in the legend of Figure 2. (PDF) [file pgen.1002529.s005.pdf]

	Mean transfer frequency (±sem)
**DNA transferred**	
pGDT4^{T}	3.2(±1)x10^{-5}
irp2^{k}	2(±0.3)x10^{-8}
**Recipient strains**	
637-Nal^{R}	1.5(±0)x10^{-5}
953-Nal^{R}	330(±170)x10^{-5}
637c-Nal^{R}	2.3(±1.3)x10^{-5}
**Temperature (broth)**	
4°C	330(±170)x10^{-5}
28°C	≤ 10^{10}
37°C	≤ 10^{10}
**Temperature (agar)**	
4°C	340(±10)x10^{-5}
28°C	≤ 10^{10}
37°C	≤ 10^{10}
