# Supplementary material for: A Natural System of Chromosome Transfer in Yersinia pseudotuberculosis
Source: PLoS Genet. 2012 Mar 8;8(3):e1002529. doi: 10.1371/journal.pgen.1002529 (PMC3297565; doi:10.1371/journal.pgen.1002529)
Supplement: Table S4 — Annotation of the putative open reading frames of pGDT4. (PDF) [file pgen.1002529.s006.pdf]

Locus tag	Start	Stop	Strand	Product		GI	Closest homolog GI
pGDT4_0001	79	3648	+	DNA primase		218534616	238755368
pGDT4_0002	3657	4088	+	TraL protein		218534617	238755368
pGDT4_0003	4133	4816	+	TraM protein		218534618	238899378
pGDT4_0004	4830	5822	+	TraN protein		218534619	238898136
pGDT4_0005	5826	7142	+	TraO protein		218534620	238899376
pGDT4_0006	7160	7960	+	TraP protein		218534621	238899375
pGDT4_0007	7971	8510	+	TraQ protein		218534622	238899374
pGDT4_0008	8651	9046	+	TraR protein		218534623	238899373
pGDT4_0009	9107	9565	+	Conserved hypothetical protein		218534624	238899372
pGDT4_0010	9620	10237	+	TraT protein		218534625	238899371
pGDT4_0011	10239	13313	+	TraU protein		218534626	238899368
pGDT4_0012	13322	14521	+	TraW protein		218534627	238898141
pGDT4_0013	15126	17285	+	TraY protein		218534628	238899365
pGDT4_0014	17364	17999	+	Exclusion-determining protein		218534629	238899364
pGDT4_0015	18107	19399	+	TrbA protein		218534630	238899361
pGDT4_0016	19414	20391	+	TrbB protein		218534631	238898148
pGDT4_0017	20404	22587	+	TrbC protein		218534632	238899359
pGDT4_0018	22598	23128	+	Putative endonuclease		218534633	37518429
pGDT4_0019	23149	23466	+	Conserved hypothetical protein		218534634	218711168
pGDT4_0020	23554	23736	+	Hypothetical protein		218534635	
pGDT4_0021	24098	24670	-	Conserved hypothetical protein		218534636	320321307
pGDT4_0022	24708	26609	-	Relaxase/mobilization nuclease MobA		218534637	238898507
pGDT4_0023	26606	26929	-	Mobilization protein MobB		218534638	162958031
pGDT4_0024	27188	27511	+	MobC protein		218534639	238755391
pGDT4_0025	28057	28488	-	Conserved hypothetical protein		218534640	162958026
pGDT4_0026	28485	28790	-	Hypothetical protein		218534641	162958026
pGDT4_0027	29242	29493	-	Conserved hypothetical protein		218534642	293397481
pGDT4_0028	29640	30095	-	Antirestriction protein KlcA		218534643	111038116
pGDT4_0029	30841	31341	-	Antirestriction protein		218534644	322836388
pGDT4_0030	31943	32257	-	Putative toxin of gyrase inhibiting toxin-antitoxin system		218534645	157369798
pGDT4_0031	32260	32496	-	Putative antitoxin of gyrase		218534646	307131729
pGDT4_0032	32561	32785	-	Hypothetical protein		218534647	
pGDT4_0033	32909	32971	-	Conserved hypothetical protein		218534648	
pGDT4_0034	33211	33384	-	Hypothetical protein		218534649	
pGDT4_0035	33436	34305	-	Similar to unknown protein		218534650	320539535
pGDT4_0036	34717	34932	-	Hypothetical protein		218534651	
pGDT4_0037	34908	35264	-	Conserved hypothetical protein		218534652	167851594
pGDT4_0038	35344	35700	-	Hypothetical protein		218534653	
pGDT4_0039	35732	35983	-	Hypothetical protein	36744	218534654	
pGDT4_0040	36816	37097	-	Probable recA regulator RecX C-terminal part		218534655	336126258
pGDT4_0041	37169	38413	+	IS*Yps2* transposase, IS*110* family, no IR		218534656	271499294
pGDT4_0042	38444	38815	-	Probable recA regulator RecX N-terminal part	IS*Yps3*	218534657	336126258
pGDT4_0043	38812	40206	-	Putative SpnT protein		218534658	38638370
pGDT4_0044	40283	40867	-	IS*Yps3* resolvase, TnpR Tn*2501*		218534659	24306036
pGDT4_0045	40986	43937	-	IS*Yps3* transposase, Tn*3* family	44043	218534660	38639566
pGDT4_0046	44006	44299	-	Hypothetical protein		218534661	317493855
pGDT4_0047	44548	45714	+	Aldo/keto reductase precursor (53% identity with YPTB1052)*		218534662	317493854
pGDT4_0048	45729	46409	+	Two component heavy metal response transcriptional regulator (84% identity with YPTB1993)*		218534663	317493853
pGDT4_0049	46425	47882	+	Putative histidine protein kinase sensor (Putative two-component system, 74% identity with YPTB1992)*		218534664	317493852
pGDT4_0050	47947	48426	+	Cupin region precursor		218534665	317493849
pGDT4_0051	48509	49162	+	Putative carboxymuconolactone decarboxylase		218534666	317493848
pGDT4_0052	49175	49759	+	Flavodoxin putative precursor		218534667	317493847
pGDT4_0053	49867	50931	+	Putative exported protein precursor (79% identity with YPTB1989)*		218534668	317493846
pGDT4_0054	51177	51296	+	Putative aldo/keto reductase, N-terminal part		218534669	218549987
pGDT4_0055	51296	51924	+	Putative aldo/keto reductase, C-terminal part		218534670	215485409
pGDT4_0057	52047	52373	-	Putative LysR-family transcriptional regulatory protein, N-terminal part		218534671	317493843
pGDT4_0058	52414	52997	-	IS*Yps1* transposase 808 bp, IR 16 bp		218534672	238756683
pGDT4_0059	53608	58068	+	Ig-like domain protein (53% identity with YPTB3789)*		218534673	153949670
pGDT4_0060	58488	59071	+	IS*Yps1* transposase 808 bp, IR 16 bp		218534674	238756683
pGDT4_0061	59109	59414	-	Putative resolvase		218534675	262042212
pGDT4_0062	59897	60520	+	Plasmid partition protein ParF		218534676	165975404
pGDT4_0063	60571	60798	+	Plasmid partition protein ParG		218534677	312174435
pGDT4_0064	61186	61769	+	IS*Yps1* transposase 808 bp, IR 16 bp		218534678	238756683
pGDT4_0065	61874	62053	+	Resolvase, C-terminal part		218534679	153001061
pGDT4_0066	62120	63040	+	Transposase of ISPlu15 subgroup, ISNCY orphans , ISPlu15, no IR no DR (72% identity with YPTB0072)*		218534680	49658869
pGDT4_0067	63174	63707	+	Hypothetical protein		218534681	162958001
pGDT4_0068	63720	64283	+	Putative IS*L3* transposase family, variable IR and DR		218534682	111038076
pGDT4_0069	64472	64762	+	Conserved hypothetical protein		218534683	116254486
pGDT4_0070	65086	65541	+	Transposase IS*Yps1-tr* (truncated)	65636	218534684	218534672
pGDT4_0071	65742	68693	+	Transposase IS*Yps3*, Tn*3* family		218534685	38639566
pGDT4_0072	68812	69396	+	Resolvase Tn*2501* family		218534686	24306036
pGDT4_0073	69473	70867	+	Putative SpnT protein	IS*Yps3*	218534687	38638370
pGDT4_0074	70864	71235	+	Probable *recA* regulator RecX, N-terminal part		218534688	336126258
pGDT4_0075	71266	72510	-	IS*Yps2* transposase, IS*110* family, no IR		218534689	271499294
pGDT4_0076	72582	72863	+	Probable *recA* regulator RecX, C-terminal part	72935	218534690	336126258
pGDT4_0077	73116	73439	+	Putative to unknown protein		218534691	157370037
pGDT4_0078	73828	74037	-	Hypothetical protein		218534692	317053280
pGDT4_0079	74342	74533	+	Hypothetical protein		218534693	

pGDT4_0080	74692	74832	-	Hypothetical protein	218534694	
pGDT4_0081	74944	75981	-	Replication initiation protein	218534695	28373019
pGDT4_0082	76979	77269	+	Conserved hypothetical protein	218534696	51593944
pGDT4_0083	77696	78154	+	Putative transcriptional activator	218534697	295885458
pGDT4_0084	78262	78510	+	Hypothetical protein	218534698	
pGDT4_0085	78529	78729	+	Hypothetical protein	218534699	238755354
pGDT4_0086	78802	79866	+	PilL protein	218534700	238755355
pGDT4_0087	80388	81164	+	Hypothetical protein	218534701	293386496
pGDT4_0088	81273	81707	+	PilM protein	218534702	38201736
pGDT4_0089	81723	83354	+	PilN protein	218534703	38201735
pGDT4_0090	83358	84656	+	PilO protein	218534704	238755358
pGDT4_0091	84646	85245	+	PilP protein	218534705	38201733
pGDT4_0092	85253	86746	+	PilQ protein	218534706	38201732
pGDT4_0093	86743	87864	+	PilR protein	218534707	238755361
pGDT4_0094	87928	88515	+	PilS protein	218534708	238755362
pGDT4_0095	88585	89076	+	PilT protein	218534709	238898127
pGDT4_0096	89073	89753	+	PilU protein	218534710	38201728
pGDT4_0097	89750	91147	+	PilV protein	218534711	238755365
pGDT4_0098	91198	91653	+	TraH protein	218534712	238755366
pGDT4_0099	91650	92438	+	TraI protein	218534713	238898515
pGDT4_0100	92380	93609	+	TraJ protein	218534714	238899381
pGDT4_0101	93641	93901	+	TraK protein	218534715	238898133
pGDT4_0102	93981	94463	+	Conserved hypothetical protein	218534716	

Lines highlighted in dark gray indicate the various transposases carried by pGDT4.

*: Identity with proteins encoded by the *Y. pseudotuberculosis* IP32953 genome.
