# Supplementary material for: A Natural System of Chromosome Transfer in Yersinia pseudotuberculosis
Source: PLoS Genet. 2012 Mar 8;8(3):e1002529. doi: 10.1371/journal.pgen.1002529 (PMC3297565; doi:10.1371/journal.pgen.1002529)
Supplement: Table S5 — ISYps1 transposition and cointegrate formation frequencies. (PDF) [file pgen.1002529.s007.pdf]

Donor	Recipient	
**IS*Yps1* transposition assay**		**[Cm^R] fraction of the recipient (±sem)[1]**
ω7249(pSW*Yps1.1*) [Cm^R]	ω4826 pir+	3.4(±0.9)x10^{-3}
ω7249(pSW*Yps1.1*) [Cm^R]	ω4826	8.5(±0.4) x10^{-6}
**Cointegrate formation assay**		**[Cm^R] fraction of the [Tmp^R] recipient (±sem)[1]**
pi3(R388, pSW23)	ω4826	<5 x10^{-8}
pi3(R388, pSW*Yps1.2*)	ω4826	9(±3)x10^{-5}
		**[Tmp^R] fraction of the recipient (±sem)[1]**
pi3(R388, pSW*Yps1.2*)	ω4826	2.2(±0.7)x10^{-1}

1. Mean of three independent experiments
